# Anatomical landmarks for localisation of the anterior ethmoidal artery: a combined radiological and cadaveric (endoscopic) study

**DOI:** 10.1007/s00276-023-03122-x

**Published:** 2023-03-20

**Authors:** Livashin Naidu, Lindokuhle A. Sibiya, Okikioluwa S. Aladeyelu, Carmen O. Rennie

**Affiliations:** 1 Discipline of Clinical Anatomy, School of Laboratory Medicine and Medical Sciences, Nelson R. Mandela School of Medicine, College of Health Sciences, University of KwaZulu-Natal, Private Bag X54001, Durban, 4001 KwaZulu-Natal South Africa; 2 Discipline of Otorhinolaryngology-Head and Neck Surgery, School of Clinical Medicine, College of Health Sciences, Nelson R. Mandela School of Medicine, University of KwaZulu-Natal, Durban, 4001 KwaZulu-Natal South Africa; 3 Present Address: Department of Human Biology and Integrated Pathology, School of Medicine, Faculty of Health Sciences, Nelson Mandela University–Missionvale Campus, Gqeberha, 6059 Eastern Cape South Africa

**Keywords:** Anterior ethmoidal artery, Anterior nasal spine, Skull base, Middle turbinate axilla, Nasal axilla, Surgical landmarks

## Abstract

**Purpose:**

The anterior ethmoidal artery is a major surgical landmark that is susceptible to iatrogenic injury during surgery of the anterior ethmoidal sinus, frontal sinus, and skull base. The present study aimed to define the location of the anterior ethmoidal artery in relation to specific anatomical landmarks using radiological imaging and endoscopic dissection.

**Methods:**

Eighty-six anterior ethmoidal arteries were assessed using computed tomography scans (bilateral analyses) and forty anterior ethmoidal arteries were assessed using cadaveric specimens (bilateral analyses). The skull base, anterior nasal spine, anterior axilla of the middle turbinate, and nasal axilla were morphometrically analysed to determine their reliability as anterior ethmoidal artery landmarks.

**Results:**

Distances to the skull base, anterior nasal spine, and nasal axilla displayed statistically significant differences between sexes and sides (*p* < 0.05). All landmarks demonstrated excellent reliability as anatomical landmarks for the localisation of the anterior ethmoidal artery, radiologically and endoscopically (ICC values ranged from 0.94 to 0.99).

**Conclusion:**

The middle turbinate axilla was the most reliable landmark, due to the lack of statistically significant differences according to sex and laterality, and the high inter-rater agreement between measurements. Anatomical knowledge of variations and relationships observed in the present study can be applied to surgeries of the anterior ethmoidal sinus, frontal sinus, and skull base to improve localisation of the anterior ethmoidal artery, preoperatively and intraoperatively, and avoid iatrogenic injury of the vessel.

## Introduction

The anterior ethmoidal artery (AEA) originates from the ophthalmic artery in the orbit [7]. It passes between the superior oblique and medial rectus eye muscles before leaving the orbit via the anterior ethmoidal foramen (situated in the fronto-ethmoidal suture) [7, 14]. The anterior ethmoidal foramen opens into the anterior ethmoidal canal, which transmits the artery through the anterior ethmoidal sinus/complex [2, 7, 14]. The artery crosses the complex at the level of its roof (skull base [SB]) or below this level, by as much as 5 mm, in a mesentery or thin bony lamella [14]. It traverses the roof/ethmoidal complex anteromedially, before entering the anterior cranial fossa (olfactory fossa) via the lateral lamella of the cribriform plate (LLCP) or the point at which the frontal bone connects to the LLCP [2, 14]. The AEA then turns anteriorly, forming a groove in the LLCP known as the anterior ethmoidal sulcus, to reach the nose via the cribriform plate [14]. The AEA supplies the frontal and ethmoidal sinuses, as well as the roof of the nose and nasal septum [7].

Functional endoscopic sinus surgery (FESS) is one of the most commonly performed procedures by otorhinolaryngologists [5]. Advancements in endoscopic technologies, instrumentation, and imaging modalities have allowed FESS to be applied not only to the nasal cavity and paranasal sinuses but also to the orbit and SB [5]. The AEA is an important landmark in FESS, used to locate the frontal sinus, frontal recess, and anterior SB [1, 5, 11]. During frontal recess surgeries, the AEA marks the posterior border of the recess [3]. Localisation of the AEA is important for frontal FESS, particularly during preoperative radiological evaulations [13]. However, the variable location of the artery complicates endoscopic surgery of the frontal recess [9]. In external approaches, identification of the AEA in the fronto-ethmoidal suture marks the anterior border of the anterior cranial fossa [3]. Localisation of the vessel also aids surgical management of anterior naso-septal perforations through the utilisation of unilateral mucosal flaps based on the AEA and its branches [4]. Additionally, its identification helps to define and treat cases of severe epistaxis and it serves as a useful landmark for the endoscopic drainage of orbital abscesses and evacuation of orbital haematomas [5, 11].

The AEA is a major anatomical landmark that is susceptible to accidental injury during surgery of the frontal sinus, frontal recess, anterior ethmoidal sinus, and SB [5, 17]. Its deep situation, complicated relations (with vital structures such as the lamina papyracea, SB, olfactory fossa, and frontal recess), and extensive variations make it a high-risk territory for surgeons [5]. A preoperative computed tomography (CT) scan is necessary to evaluate the complex anatomy of the AEA [1]. The AEA displays significant variability as it traverses the anterior ethmoidal sinus from the orbit to the LLCP [1]. Its position may even vary on either side of the same individual [11]. The segment of the AEA that traverses the anterior ethmoidal sinus is the most vulnerable during surgery, hence surgeons need to be aware of variations [11, 16]. Detailed anatomical knowledge pertaining to the AEA and its possible variations is crucial to avoid complications during surgery [1]. Iatrogenic injury of the AEA could result in intra-orbital bleeding, profuse epistaxis, a retro-orbital haematoma (that may lead to blindness if not decompressed within one hour), intracranial bleeding (in rare cases), and cerebrospinal fluid leaks [1, 5, 11, 17].

Many studies have provided guidelines to better facilitate AEA identification and localisation preoperatively and during surgical procedures [5, 6, 9, 10, 17]. Preoperative identification of the AEA and its variations on CT scans reduces the risk of iatrogenic injury and is important for safe and effective FESS [5, 11]. Knowledge of anatomical landmarks that can be utilised intraoperatively is also important, to facilitate dissection in the AEA territory [17]. Anatomical variations of the AEA observed between different studies, including those related to landmarks for its accurate and precise localisation, could be due to differences between population groups [6]. Information concerning reliable AEA landmarks in the South African population is scarce in the literature.

The present study aimed to define the location of the AEA in a South African population in relation to specific anatomical landmarks using radiological imaging and endoscopic dissection.

## Materials and methods

The present study comprised a retrospective review of 86 AEAs using CT scans (radiological subset) and an observational analysis of 40 AEAs using cadaveric (endoscopic) dissection (cadaveric subset). Forty-three CT scans of adult individuals (28 males; 15 females) and twenty embalmed adult cadaveric heads (13 males; 7 females) were analysed, with left and right sides being assessed separately in both subsets (bilateral analysis). The CT scans were obtained from Inkosi Albert Luthuli Central Hospital (IALCH) in KwaZulu-Natal. The cadaveric heads were obtained from the Discipline of Clinical Anatomy, School of Laboratory Medicine and Medical Sciences, College of Health Sciences at the University of KwaZulu-Natal (UKZN). The mean age of the patients in the CT cohort was 37.6 years old, ranging between 18 and 88 years of age, and that of the cadavers was 78.8 years old, ranging between 67 and 91 years of age. The CT cohort comprised Black African (83.7%), Indian (11.6%) and Coloured (4.7%) patients. All cadavers were White individuals (100%). Ethical clearance for this study was obtained from the Biomedical Research Ethics Committee (BREC) at UZKN (BREC/00001852/2020). Permission to access CT scans and conduct this research at IALCH was sought and approved by the institution (Reference: 21312101) and the KwaZulu-Natal Department of Health (NHRD Ref: KZ_202010_007). Permission to utilise the cadavers and conduct this research at UKZN was sought and approved by the institution. The inclusion criteria for scan and cadaver selection were as follows: patients 18 years of age and older, scans without observable evidence of previous surgery or pathology, trauma and distortion affecting the nasal cavity, frontal sinus, and anterior ethmoidal sinus, and with a slice thickness of 1 mm or less, embalmed adult cadaveric heads with no previous injuries, fractures, surgeries or any other macroscopic evidence of pathology and that have not been previously dissected in the region of the nasal cavity, ethmoidal sinus and frontal sinus.

**Fig. 1:**
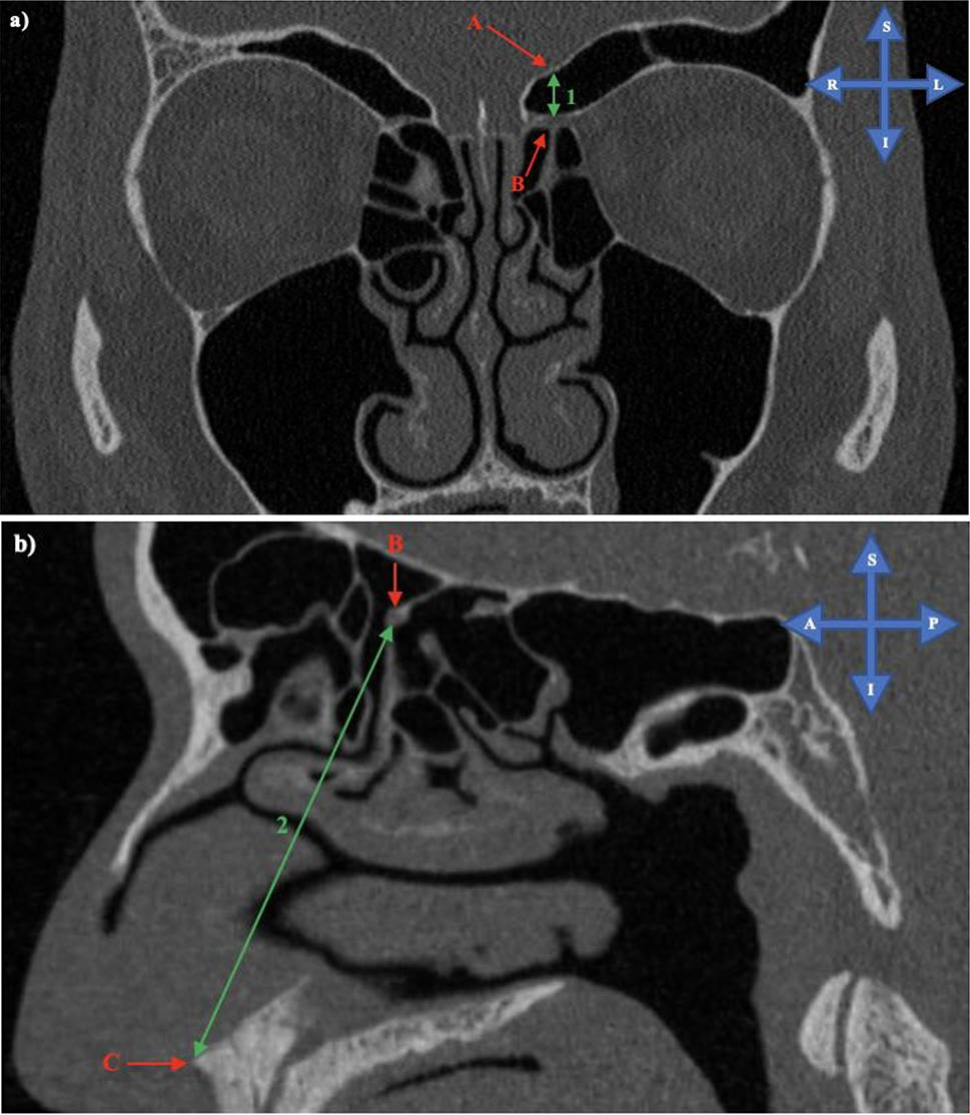
**a** Measurement of the vertical distance from the anterior ethmoidal artery to the skull base on the coronal plane and, **b** measurement of the distance from the anterior ethmoidal artery to the anterior nasal spine on the oblique sagittal plane. Key: A = Skull base, B = Anterior ethmoidal artery, C = Anterior nasal spine, 1 = Vertical distance between the anterior ethmoidal artery and skull base, 2 = Distance between the anterior ethmoidal artery and anterior nasal spine, *S* superior, *I* inferior, *R* right, *L* left, *A* anterior, *P* posterior

The CT scans were acquired by IALCH during clinical routines with either a 128-slice SOMATOM Definition AS Scanner or SOMATOM Definition Flash CT Scanner (Siemens Healthineers, Forcheim, Germany) and saved as digital imaging and communications in medicine (DICOM) files. The DICOM images were viewed and analysed at IALCH using *syngo*.plaza software (version VB20A), the standard software used by this medical institution. The distance from the midpoint of the AEA (intranasal portion) to the SB and anterior nasal spine (ANS) was measured and analysed (Fig. 1). The vertical distance from the AEA to the SB was measured using coronal images (Fig. 1a). To determine the distance between the AEA and ANS, oblique sagittal images were reconstructed from the respective coronal images. An axis that passed through the AEA and the midline was first angled in the coronal plane. The measurements were then taken from the oblique sagittal plane (Fig. 1b). The distance to the SB was classified into three groups: attached to the SB or < 2.5 mm (Group1), ≥ 2.5 mm, and ≤ 5 mm (Group 2), > 5 mm (Group 3).

**Fig. 2:**
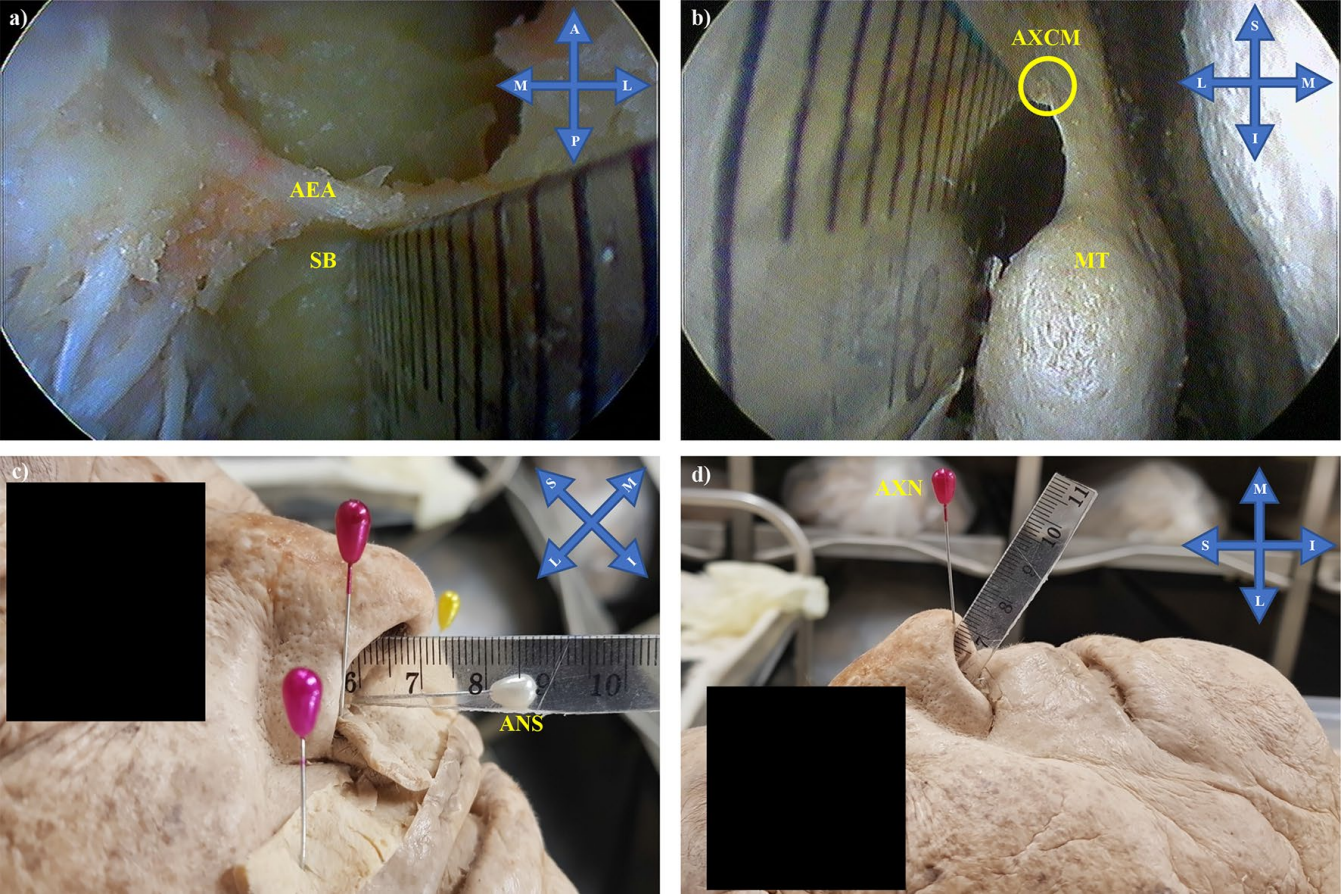
**a** Measurement of the distance from the anterior ethmoidal artery (AEA) to the skull base (SB), **b** Measurement of the distance from the anterior ethmoidal artery (situated deeper) to the anterior axilla of the middle turbinate (AXCM), **c** Measurement of the distance from the anterior ethmoidal artery (situated within the nasal cavity) to the anterior nasal spine (ANS – white pin), and d) Measurement of the distance from the anterior ethmoidal artery (situated within the nasal cavity) to the nasal axilla (AXN – pink pin) Key: *S* Superior, *I* inferior, *M* medial, *L* lateral, *MT* middle turbinate

The AEA was endoscopically dissected bilaterally in the embalmed adult cadaveric heads using a 4 mm, 0° Rudolf endoscope and appropriate instruments. The endoscopic dissection comprised of uncinectomy and anterior ethmoidectomy (via the nose), until the AEA was identified near the anterior SB. The lamina papyracea was then removed anteriorly and adjacent to the AEA. Finally, the lamina papyracea and the periorbital tissue were detached to confirm the AEA identification in the region where it penetrated the lamina papyracea, via the anterior ethmoidal foramen. The distance from the midpoint of the AEA (intranasal portion) to the SB, ANS, anterior axilla of the middle turbinate (AXCM) (anterior border of the middle turbinate insertion on the lateral nasal wall), and nasal axilla (AXN) (superomedial nostril border—region where the lateral and medial inferior lateral cartilage crura meet) was then measured and analysed after these landmarks had been identified (Fig. 2). Intranasal measurements were taken using a 110 mm-long and 10 mm-wide plastic ruler that was thin enough for insertion into the middle meatus. The distance to the SB was classified into three groups: attached to the SB or < 2.5 mm (Group1), ≥ 2.5 mm and ≤ 5 mm (Group 2), > 5 mm (Group 3).

Each measurement was repeated three times by the principal investigator and the intra-observer error was determined through analysis of the three measurements. The measurements from 25% of CT scans and cadavers were repeated three times by a second observer to determine the inter-observer error.

### Statistical analysis

All data were summarised using descriptive statistics (frequencies and percentages or means, standard deviations, medians, interquartile ranges, and ranges). Morphometrical data pertaining to each anatomical landmark of interest were analysed and compared according to sex, laterality, and modality (when applicable) using t-tests or Wilcoxon Rank Sum/Mann–Whitney U tests, as appropriate. T-tests were utilised if data displayed a normal distribution and Wilcoxon Rank Sum tests/Mann–Whitney U tests were utilised if data displayed a non-normal distribution. Configurations of SB groupings were compared according to sex and laterality using Fisher’s exact tests or McNemar’s tests, as appropriate. Intra-observer and inter-observer errors were calculated and represented as intraclass correlation coefficient (ICC) values to determine the reliability of the morphometrical data. All data were analysed using *R* Statistical Computing Software of the *R* Core Team version 3.6.3. A *p*-value of less than 0.05 was considered statistically significant.

## Results

### Intra- and inter-observer error

Intra- and inter-observer error analyses yielded ICC values ranging from 0.94 to 0.99 on the right and 0.95 to 0.99 on the left for the four landmarks assessed, indicating that all landmarks display excellent reliability for localisation of the AEA. The AXCM and AXN displayed excellent reliability as endoscopic landmarks, while the SB and ANS displayed excellent reliability as both radiological and endoscopic landmarks.

### Radiological subset

The overall median (Q1–Q3) distance from the AEA to the a) SB was 2.40 (0–3.60) mm, and b) ANS was 55.0 (53.4–58.1) mm. Significant differences in the measured parameters were identified in the distance from the AEA to the a) SB in terms of laterality (*p* < 0.001), and b) ANS in terms of sex (*p* < 0.001) and laterality (*p* < 0.001). The distances to the SB were found to be greater on the right side, and those to the ANS were greater in males and on the left side (Table 1).

**Table 1:** Distance from the anterior ethmoidal artery to each radiological landmark

	Skull base (mm)		*p*-value	Anterior nasal spine (mm)		*p*-value
	Median (Q1–Q3)^a^	Range		Median (Q1–Q3)^a^	Range	
Overall	2.40 (0–3.60)	0–8.20	–	55.0 (53.4–58.1)	46.9–62.4	–
Right	1.90 (0–3.00)	0–8.20	< 0.001*	54.7 (53.1–57.9)	46.9–61.9	< 0.001*
Left	2.90 (0–3.90)	0–6.90		55.6 (53.7–58.7)	48.3–62.4	
Male	2.35 (0–3.10)	0–8.20	0.185	57.3 (54.6–59.6)	51.1–62.4	< 0.001*
Female	2.70 (0–3.88)	0–5.30		53.5 (50.8–54.5)	46.9–56.8	

^a^Median (Q1–Q3) presented instead of mean ± SD, due to the non-normal distribution of the data

^*^Statistically significant (*p* < 0.05)

**Fig. 3:**
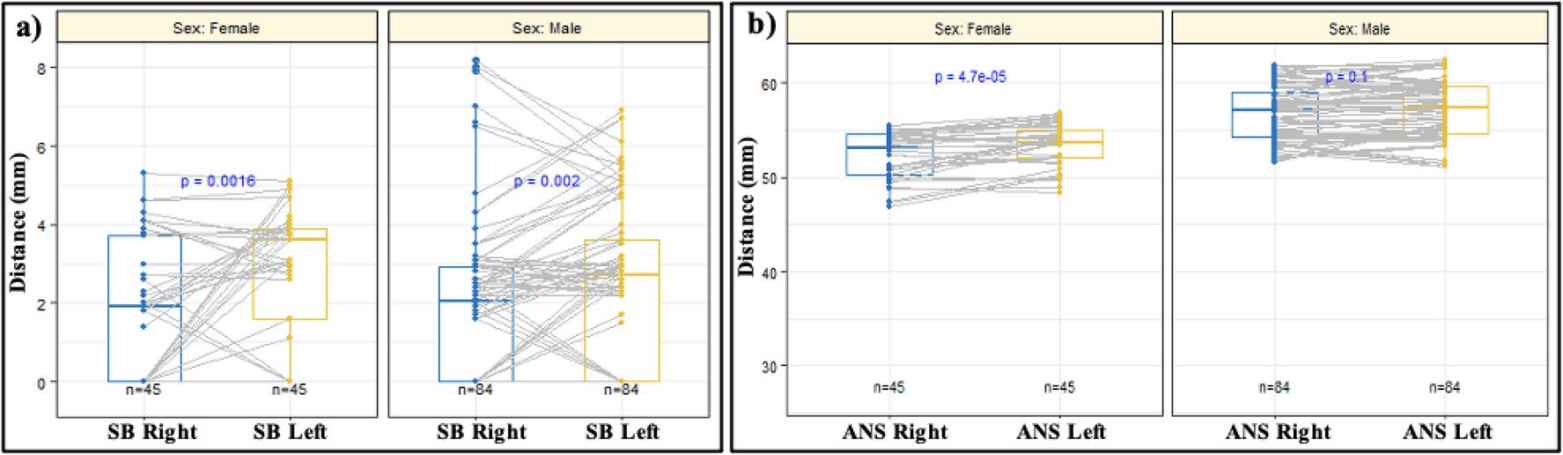
Box plots illustrating the comparison of the distance to the **a** skull base (SB), and **b** anterior nasal spine (ANS), between the right and left sides of male individuals, and the right and left sides of female individuals

Furthermore, there were statistically significant differences in the distance to the SB between laterality for both males and females (*p* = 0.002 & *p* = 0.0016, respectively), with greater distances observed on the left sides (Fig. 3). The distance to the ANS showed statistically significant differences between right sides of males and females, and between left sides of males and females (*p* < 0.001), with both sides greater in males (Table 2). All other comparisons displayed no statistically significant differences (Tables 1 and 2; Fig. 3).

**Table 2:** Distribution of the distance from the anterior ethmoidal artery to each anatomical landmark in the radiological subset, according to sex and laterality

	Male		Female		*p*-value
	Median (Q1–Q3)^a^	Range	Median (Q1–Q3)^a^	Range	
Skull base (mm)					
Right	2.05 (0–2.93)	0–8.20	1.90 (0–3.70)	0–5.30	0.994
Left	2.70 (0–3.60)	0–6.90	3.60 (1.60–3.90)	0–5.10	0.079
Anterior Nasal Spine (mm)					
Right	57.2 (54.3–59.0)	51.6–61.9	53.1 (50.2–54.5)	46.9–55.4	< 0.001*
Left	57.4 (54.6–59.6)	51.1–62.4	53.7 (52.0–55.0)	48.3–56.8	< 0.001*

^**a**^Median (Q1–Q3) presented instead of mean ± SD, due to the non-normal distribution of the data

^*^Statistically significant (*p* < 0.05)

#### Skull base groups

**Fig. 4:**
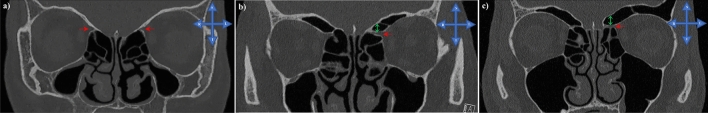
**a** Skull base group 1 artery on both sides (attached to the skull base), **b** Skull base group 2 artery on the left side, and **c** Skull base group 3 artery on the left side (coronal planes). Key: Red arrow = Anterior ethmoidal artery, Green arrow = Vertical distance between the anterior ethmoidal artery and the skull base, *S* superior, *I* inferior, *R* right, *L* left

Group 1 arteries were observed most frequently (53.5% of cases), followed by Group 2 arteries (39.5% of cases) (Table 3; Fig. 4). Group 3 arteries were seen least frequently (7% of cases) (Table 3; Fig. 4). Statistically significant differences in SB groups were noted in terms of laterality (*p* = 0.039) (Table 3). Group 1 arteries were more common on the right side, while Group 2 and 3 arteries were more common on the left (Table 3). However, no significant differences were noted in terms of sex (*p* = 0.052) (Table 3).

**Table 3:** Classification of the distance from the anterior ethmoidal artery to the skull base in the radiological subset

	Group 1 *n* (%)	Group 2 *n* (%)	Group 3 *n* (%)	Total *n* (%)	*p*-value
Right	29 (67.4)	12 (27.9)	2 (4.7)	43 (100)	0.039*
Left	17 (39.5)	22 (51.2)	4 (9.3)	43 (100)	
Male	32 (57.1)	18 (32.1)	6 (10.7)	56 (100)	0.052
Female	14 (46.7)	16 (53.3)	0 (0)	30 (100)	
Total	46 (53.5)	34 (39.5)	6 (7.0)	86 (100)	–

^*^Statistically significant (*p* < 0.05)

### Cadaveric subset

The overall median (Q1–Q3) distance from the AEA to the SB was 0 (0–2.00) mm (Table 4). The overall mean distance from the AEA to the a) ANS was 59.3 ± 5.12 mm (mean ± standard deviation [SD]), b) AXCM was 20.9 ± 3.65 mm, and c) AXN was 69.3 ± 5.20 mm (Table 4). Statistically significant differences between the two sexes were identified in the distance to the ANS (p < 0.001) and AXN (*p* < 0.001), with males displaying greater distances than females (Table 4). Furthermore, statistically significant differences, in these distances, were identified between the right sides of males and females (*p* < 0.001 for both landmarks) and between the left sides of males and females (*p* = 0.010 and *p* < 0.001, respectively) (Table 5). Males consistently displayed greater distances than females (Table 5). All other comparisons displayed no statistically significant differences (*p* > 0.05) (Tables 4 and 5).

**Table 4:** Distance from the anterior ethmoidal artery to each cadaveric landmark

	Skull base (mm)		*p*-value	Anterior nasal spine (mm)		*p*-value
	Median (Q1–Q3)^a^	Range		Mean ± SD	Range	
Overall	0 (0–2.00)	0–5.00	–	59.3 ± 5.12	48.0–71.0	–
Right	0 (0–2.00)	0–5.00	0.97	59.5 ± 4.78	48.0–69.0	0.51
Left	0 (0–2.00)	0–5.00		59.2 ± 5.47	50.0–71.0	
Male	0 (0–2.00)	0–5.00	0.238	60.7 ± 4.68	50.5–71.0	< 0.001*
Female	0.5 (0–2.00)	0–5.00		56.7 ± 4.91	48.0–65.0	

	Anterior axilla of the middle turbinate (mm)		*p*-value	Nasal axilla (mm)		*p*-value
	Mean ± SD	Range		Mean ± SD	Range	
Overall	20.9 ± 3.65	13.0–31.0	–	69.3 ± 5.20	57.0–81.0	–
Right	20.7 ± 3.61	14.0–30.0	0.42	69.3 ± 4.70	60.0–77.0	0.92
Left	21.2 ± 3.70	13.0–31.0		69.3 ± 5.70	57.0–81.0	
Male	21.1 ± 3.34	14.0–31.0	0.444	71.2 ± 4.11	63.0–81.0	< 0.001*
Female	20.6 ± 4.18	13.0–30.0		65.8 ± 5.24	57.0–77.0	

*Statistically significant (*p* < 0.05)

^a^Median (Q1–Q3) presented instead of mean ± SD, due to the non-normal distribution of the data

**Table 5:** Distribution of the distance from the anterior ethmoidal artery to each anatomical landmark in the cadaveric subset, according to sex and laterality

	Male		Female		*p*-value
	Mean ± SD	Range	Mean ± SD	Range	
Skull base (mm)					
Right	0 (0–1.50)^**a**^	0–5.00	2.00 (0–2.00)^**a**^	0–5.00	0.062
Left	0 (0–2.00)^**a**^	0–5.00	0 (0–3.00)^**a**^	0–4.00	0.885
Anterior nasal spine (mm)					
Right	61.0 ± 3.77	54.0–69.0	56.6 ± 5.27	48.0–65.0	< 0.001*
Left	60.5 ± 5.48	50.5–71.0	56.7 ± 4.64	50.0–65.0	0.010*
Anterior axilla of the middle turbinate (mm)					
Right	20.0 (18.3–23.0)^**a**^	17.0–27.0	20.0 (17.0–23.0)^**a**^	14.0–30.0	0.523
Left	21.5 ± 3.77	14.0–31.0	20.6 ± 3.59	13.0–25.0	0.385
Nasal axilla (mm)					
Right	71.2 ± 3.57	64.0–77.0	65.9 ± 4.62	60.0–73.0	< 0.001*
Left	71.2 ± 4.63	63.0–81.0	65.8 ± 5.91	57.0–77.0	< 0.001*

^*^Statistically significant (*p* < 0.05)

^**a**^Median (Q1–Q3) presented instead of mean ± SD, due to the non-normal distribution of the data

#### Skull base groups

**Fig. 5:**
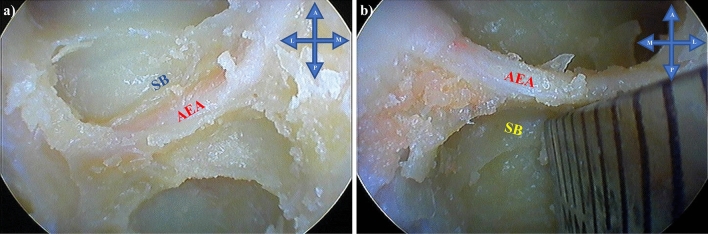
**a** Skull base group 1 artery (attached to the skull base) and **b** Skull base group 2 artery (≥ 2.5 mm and ≤ 5 mm from the skull base). Key: *AEA* anterior ethmoidal artery, *SB* skull base, *A* anterior, *P* posterior, *L* lateral, *M* medial

Group 1 arteries were observed most frequently (82.5% of cases), followed by Group 2 arteries (17.5% of cases) (Fig. 5). No Group 3 arteries or statistically significant differences according to sex (*p* = 0.679) or laterality (*p* = 0.317) were observed.

### Modality comparison: radiological subset vs cadaveric subset

**Fig. 6:**
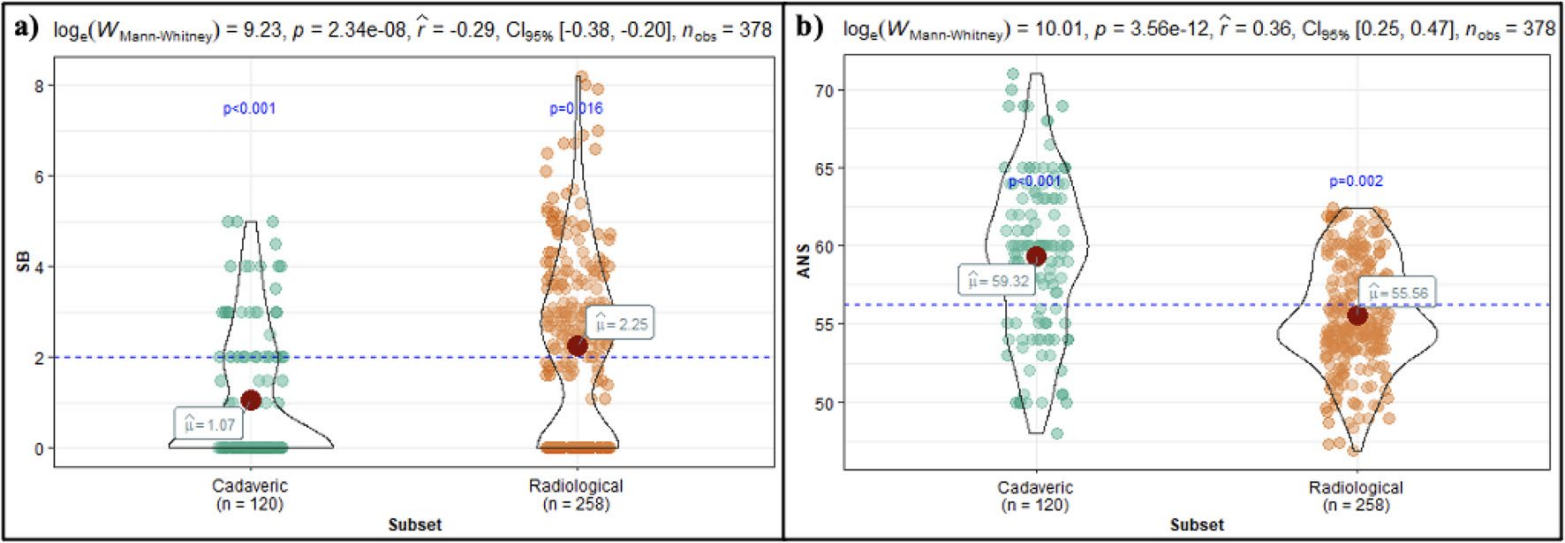
Violin plots illustrating the comparison of the overall distance to the **a** skull base (SB), and **b** anterior nasal spine (ANS), between the radiological and cadaveric subsets

Statistically significant differences between the two subsets were identified in the overall distance to the SB (*p* < 0.001) and ANS (*p* < 0.001), with the radiological subset displaying greater distances to the SB and the cadaveric subset displaying greater distances to the ANS (Fig. 6). Significant differences were also identified in the distance to the SB and ANS among individual sides and sexes (*p* < 0.001 in all cases). The radiological subset always displayed greater distances to the SB, while the cadaveric subset always displayed greater distances to the ANS.

## Discussion

The present study observed similar distances to the SB, ANS and AXCM in the radiological and cadaveric subsets when compared to earlier literature [3, 6, 10, 13, 15, 17]. However, higher results were recorded in the distance to the a) SB in females within the radiological subset, and b) AXN (overall and in terms of sex and side) as compared to the literature [3, 5, 6, 10, 13]. Moreover, lower results were recorded in the distance to the a) SB in the cadaveric subset (overall and in terms of sex and side), and b) ANS in both sexes within the radiological subset [1, 3, 5, 6, 9–11].

Earlier literature has identified statistically significant differences in the distance to the ANS and AXN according to sex with males displaying greater distances than females, however, no significant differences have been identified according to laterality [6, 10, 15]. In addition, the distances to the SB and AXCM have displayed no statistically significant differences according to sex or laterality in the literature [3, 5, 6, 8, 10, 17]. Results from the present study’s cadaveric subset support the findings of the literature in respect of all four landmarks.

In contrast, the radiological subset disagrees with the literature in part, having displayed no significant difference in the distance to the SB according to sex (*p* = 0.185), but a significant difference according to laterality (*p* < 0.001: greater distances observed on the right side), and significant differences in the distance to the ANS according to both sex and laterality (*p* < 0.001: greater distances observed in males and on the left side). The greater distances to the ANS and AXN displayed by male individuals can be attributed to the morphological differences between male and female skulls (in general, male skulls are more robust and female skulls more gracile) and noses (“the female nose is slightly smaller and narrower than the male nose”) [7]. Laterality differences in the distance to the SB and ANS are likely due to slight skeletal differences in the SB and the variable position of the AEA on either side (the ANS is a midline structure, that is, its position is constant irrespective of the side being analysed).

The AEA’s course as it passes through the ethmoidal sinus varies according to its relationship with the SB [1]. Filho et al*.* [6] and Joshi et al*.* [11] (in the absence of a supraorbital ethmoid cell) have previously recorded Group 1 arteries most frequently in their studies. Wormald et al*.* [20] defined a supraorbital ethmoid cell as “an anterior ethmoid cell that pneumatizes around, anterior to, or posterior to the anterior ethmoidal artery over the roof of the orbit”. The present study’s cadaveric subset further corroborates these results. Although, the radiological subset displayed a much lower frequency of Group 1 arteries and a subsequent higher frequency of Group 2 arteries as compared to previous studies [6, 11]. In contrast to previous literature, no Group 3 arteries were observed in the present study’s cadaveric subset [6, 11]. Filho et al*.* [6] identified no statistically significant difference regarding SB groups between the two sides (*p* = 0.383). The present study’s cadaveric subset agrees with this finding. However, results obtained from the radiological subset differ, with a significant difference according to laterality being observed (*p* = 0.039).

Filho et al*.* [6] and Lee et al*.* [13] found that the AXCM was the most reliable and useful landmark for the localisation of the AEA in clinical practice. Furthermore, Pernas et al*.* [17] stated that the AXCM was one of the two most consistent landmarks identified in their study. The lack of significant differences between sexes and sides (all *p*-values > 0.05), together with the ICC values of 0.96 on the right and 0.97 on the left, indicate that the AXCM displays excellent consistency and reliability as a landmark for the localisation of the AEA in the present study. Therefore, the present study agrees with the findings of earlier literature, identifying the AXCM as the most reliable landmark for the localisation of the AEA [6, 13, 17].

Noteworthy differences between previously studied populations and the present study’s populations (that is, the 100% White-cadaveric subset/population and the predominantly Black African-radiological subset/population) can be seen. In summary, the cadaveric subset observed shorter distances to the SB (in both sexes, either side, and overall) and greater distances to the AXN (in both sexes, either side, and overall) when compared to previous literature [1, 3, 5, 6, 9–11, 13]. While the radiological subset noted greater distances to the SB (in females only) and shorter distances to the ANS (in both sexes) in comparison to earlier studies [3, 5, 6, 10]. In addition, previous literature has documented no significant relationship between laterality and the distances to the SB and ANS [3, 5, 6, 10, 15]. However, the radiological subset noted statistically significant differences according to laterality in both distances.

Anatomical variations observed between the present study and previous studies can be due to differences between different population groups [6, 10]. Previous studies have assessed many different populations, particularly predominantly White and Asian populations [1, 3, 5, 6, 8–11, 13, 15, 17]. However, there is a paucity of information pertaining to reliable AEA landmarks in the South African population, a predominantly Black African population. To prevent unnecessary complications during endoscopic sinus surgery, surgeons need to recognise AEA variations in different populations [1]. The South African population presents a unique setting, due to its diversity of population groups: majority Black African (80.9%), Coloured (8.8%), White (7.8%), and Indian (2.6%) [18]. The population group distribution displayed by the entire South African population accounts for the distribution of population groups within the present study’s radiological subset.

The significant differences observed between the radiological and cadaveric subsets were likely due to the population differences between the two subsets (Fig. 6). The radiological subset comprised mostly Black African patients, as well as a few Indian and Coloured patients, while the cadaveric subset comprised of only White individuals. Significant variation exists between the different population groups in South Africa as a result of ecology, geography, culture, and language influencing variation in modern and historic populations [12]. In addition, morphological differences between population groups in the South African population are prevalent because of positive assortative mating and social forces acting as gene flow barriers that limit group interaction and increase variation between groups [19]. For instance, White individuals in the South African population have been shown to display long ANSs, while Black African individuals display short ANSs [12]. These documented observations between population groups support the present study’s findings, with the cadaveric subset (which comprised only White individuals) displaying greater distances to the ANS compared to the radiological subset (which comprised mostly Black African individuals). The present study’s results regarding these population differences are further corroborated by earlier literature, with findings similar to those of the cadaveric subset being noted in Western populations that comprised of mostly White individuals and findings similar to those of the radiological subset being noted in Spanish and Asian populations [6, 10, 15].

Other feasible reasons for the variations observed between the present study and earlier literature are slight differences in measurement parameters and modalities utilised (radiological or cadaveric). For instance, Han et al*.* [8] measured the antero-posterior distance between the AEA and AXCM without taking the height difference between the two structures into consideration. In addition, the present study measured the distance between the midpoint of the AEA (intranasal portion) and the landmarks of interest. Whereas previous studies have taken measurements from the midpoint of the AEA, the entry point of the AEA into the SB and the exit point of the AEA from the orbit (anterior ethmoidal foramen) [1, 6, 15]. The present study intended to compare results between the radiological and cadaveric subsets (Fig. 6). Therefore, to maintain consistency between findings, the midpoint of the AEA was utilised for measurements as it could be more easily identified and utilised in both subsets, particularly the cadaveric subset. Additional landmarks (that is, the AXCM and AXN) were examined in the cadaveric subset as these can be used to localise the AEA during endoscopic surgeries, with little to no dissection being required. Another possible reason for the disparity between the two subsets is slight differences between living tissue and embalmed tissue, due to changes tissue would undergo when embalmed (soft tissue structures specifically can experience changes—blood vessels may even collapse) [7].

Variations in measurements, no matter how minute, are still important and caution needs to be exercised when dissecting the fronto-ethmoidal region [13]. Endoscopic visualisation of crucial structures should remain the standard in determining how to proceed during FESS [13]. Preoperative identification of AEA variations assists in reducing the risk of AEA traumatisation, especially if the AEA is situated below the SB [1]. Failure to identify an AEA in a mesentery and off the SB could result in accidental injury of the artery while clearing septations at the SB during surgery [1].

## Conclusion

The present study provided updated information pertaining to anatomical landmarks for accurate localisation of the AEA. This study concludes that all four landmarks (that is, the SB, ANS, AXCM and AXN) can be reliable anatomical landmarks for the localisation of the AEA, with the AXCM being the most reliable. However, differences in terms of sex, laterality, and population groups must also be considered when utilising these landmarks. Anatomical knowledge gained from this study can be applied to surgeries of the frontal sinus, anterior ethmoidal sinus and SB (such as FESS), to improve preoperative and intraoperative localisation of the AEA, and avoid its iatrogenic injury. Limitations of this study included sample restrictions, that is, the uneven distribution of sexes and population groups across both subsets.

## Data Availability

All data generated and analysed during the current study are available from the corresponding author upon reasonable request.
